# Acceptability of patient-centered hypertension education delivered by community health workers among people living with HIV/AIDS in rural Uganda

**DOI:** 10.1186/s12889-021-11411-6

**Published:** 2021-07-07

**Authors:** Charles Batte, John Mukisa, Natalie Rykiel, David Mukunya, William Checkley, Felix Knauf, Robert Kalyesubula, Trishul Siddharthan

**Affiliations:** 1 School of Medicine, Lung Institute, Makerere University College of Health Sciences, Upper Hill Mulago Hill, Kampala, Uganda; 2 Department of Immunology and Molecular Biology, School of Biomedical Sciences, Makerere University College of Health Sciences, Kampala, Uganda; 3 Division of Pulmonary and Critical Care, School of Medicine, Johns Hopkins University, Baltimore, USA; 4 Center for Global Non-Communicable Disease Research and Training, Johns Hopkins University, Baltimore, USA; 5 Faculty of Health Sciences, Busitema University, Mbale, Uganda; 6 Department of Nephrology and Medical Intensive Care, Charité - Universitätsmedizin Berlin, Berlin, Germany

**Keywords:** Acceptability, PLWHA, Patient education, PocketDoktor™, Hypertension

## Abstract

**Background:**

The prevalence of hypertension is increasing among people living with HIV/AIDS (PLWHA) in low- and middle-income countries (LMICs). However, knowledge of the complications and management of hypertension among PLWHA in Uganda remains low. We explored the acceptability of implementing hypertension (HTN) specific health education by community health workers (CHWs) among PLWHA in rural Uganda.

**Methods:**

We conducted a qualitative study consisting of 22 in-depth interviews (14 PLWHA/HTN and 8 CHWs), 3 focus group discussions (FGDs), 2 with PLWHA/HTN and 1 with CHWs from Nakaseke district, Uganda. Participants were interviewed after a single session interaction with the CHW. Data were transcribed from *luganda* (local language) into English and analyzed using thematic analysis. We used Sekhon’s model of acceptability of health Interventions to explore participants’ perceptions.

**Results:**

Participants believed CHWs utilized easy-to-understand, colloquial, non-technical language during education delivery, had a pre-existing rapport with the CHWs that aided faster communication, and had more time to explain illness than medical doctors had. Participants found the educational material (*PocketDoktor™*) to be simple and easy to understand, and perceived that the education would lead to improved health outcomes. Participants stated their health was a priority and sought further disease-specific information. We also found that CHWs were highly motivated to carry out the patient-centered education. While delivering the education, CHWs experienced difficulties in keeping up with the technical details regarding hypertension in the *PocketDoktor™,* financial stress and patient questions beyond their self-perceived skill level and experience. PLWHA/HTN had challenges accessing the health facility where the intervention was delivered and preferred a household setting.

**Conclusions:**

Hypertension patient-centered education delivered by CHWs using the PocketDoktor™ was acceptable to PLWHA and hypertension in Nakaseke area in rural, Uganda. There is need for further studies to determine the cost implications of delivering this intervention among PLWHA across LMIC settings.

**Supplementary Information:**

The online version contains supplementary material available at 10.1186/s12889-021-11411-6.

## Background

The growing burden of non-communicable diseases (NCDs) among people living with HIV/AIDS (PLWHA) threatens to undermine the global progress that has been made to manage the HIV/AIDs epidemic in the last thirty years [1]. Hypertension (HTN) is the most common NCD and its prevalence among PLWHA has doubled over the past decade. This may be due to PLWHA living longer, increased access to antiretroviral therapy and lifestyle changes [2–4]. The long-term effects of hypertension include significant morbidity and mortality, and health outcomes may be more deleterious among PLWHA attributable to a combination of risk factors, including exposure to protease inhibitors, aging and direct consequences of HIV [1, 5–7]. In Uganda, the estimated prevalence of hypertension among adults was 26.4% in 2015 and 31.5% in 2016 [8–10]. A study among PLWHA in an ambulatory care center found the prevalence of HTN in this risk group rose from 16.9% in 2009 to 32.3% in 2013 [11]. Although HIV/AIDS programs are well established across Uganda, these have yet to fully integrate hypertension management with HIV care and PLWHA often have limited knowledge about hypertension. For instance, a study conducted in rural Wakiso district of central Uganda showed very poor knowledge about hypertension and its consequences, with over 80% of the participants unaware of their diagnosis [12].

A number of interventions have been proposed to improve HTN diagnosis and management among PLWHA including patient-centered education and task-shifting approaches [13]. Patient-centered education has previously been implemented in LMICs and is defined as “a partnership between health care providers, patients, and families that provides patients with the information necessary to participate in medical decision-making” [14]. Equipping patients with disease-specific knowledge and lifestyle behavioral change information can improve clinical outcomes for patients with hypertension [15, 16]. Studies in high-income settings have shown that patient-centered health education interventions such as monthly interactive educational workshops on hypertension, dietary planning and sodium restriction education improve blood pressure control among patients with hypertension [17, 18]. Similarly, in Uganda, the *PocketDoktor™* booklet has previously been used to implement patient centered education for chronic diseases in urban, Kampala with demonstrated increase in patient activation as well as patient satisfaction [19].

Task-shifting approaches have additionally proven successful for the management of both HIV and NCDs globally [20–23]. Community Health Workers (CHWs), a pre-existing structure within many low- and middle-income countries (LMIC) health systems, have formed the pillar of these task-shifting activities. CHWs are trained members of the community who provide health education and basic services to communities with roles varying according to country and circumstances [24, 25]. For example, in Uganda, CHWs participate in activities like immunization, community mobilization, community case management, referral of children with malaria and pneumonia symptoms, and distribution of health commodities like insecticide treated mosquito nets, de-worming tablets in some places [26]. Established in 2001, the existing network of CHWs in Uganda has successfully implemented public health interventions in diseases such as HIV, malaria and Tuberculosis (TB) [26, 27]. Because they reside in the communities they serve, CHWs provide an opportunity for communities to actively and timely engage in care and improve chronic diseases management. However, little is known about patients’ acceptability of CHWs in NCD care, particularly for dual diagnoses of HIV and NCDs.

Acceptability of healthcare interventions is a measure of how individuals participating in or providing an intervention perceive it as tolerable depending on their expected or experienced cognitive and emotional responses [28]. To assess acceptability of health interventions, Sekhon et al. proposed a theoretical framework of acceptability (TFA) with seven constructs that include affective attitude, burden, perceived effectiveness, ethicality, intervention coherence, opportunity costs, and self-efficacy [28]. We draw on these constructs to guide our presentation of findings from a qualitative study to understand the acceptability of the implementation of a patient-centered education intervention delivered by CHWs using the *PocketDoktor™* among PLWHA with hypertension in Nakaseke, a rural Ugandan setting.

## Methods

### Study design

We carried out an exploratory qualitative study using focus group discussions (FGD) and in-depth interviews (IDI) with CHWs, and PLWHA and HTN. We recruited participants between January and June 2019 at the Nakaseke antiretroviral treatment (ART) clinic, from an ongoing cohort of 2000 PLWHA.

### Study setting

Nakaseke is a rural community with 75% of inhabitants engaged in subsistence farming and 60% living on less than 45,000 Uganda shillings (USD 13) per month [11]. Nakaseke hospital has 18 CHWs attached to the health promotion department in this area. CHWs reside in the same community served by the hospital catchment area and are selected by the community leadership. The CHWs participate in health education, distribution of insecticide -treated mosquito nets, referral of sick patients, mass immunization and community mobilization for health campaigns. Two doctors and five nurses run the ART clinic with primary support from the Uganda government Ministry of Health and its HIV-related donor partners. We conducted all study interviews in private rooms at the health facility to ensure respondents were most comfortable engaging.

### Instrument development

Focus group discussion and in-depth interview guides were developed with a multidisciplinary team (medical doctor, nurses, and CHWs) guided by the Theoretical Framework of Acceptability (TFA) construct. A semi-structured FGD interview guide was used for each participant group, all of which underwent an iterative process with modifications made throughout the pilot phase of the study [12]. The questions examined the customs, perceptions and beliefs related to HTN care provided by the CHWs. The guides consisted of open-ended questions with embedded prompts to probe the study participants to express their views. The same questions were administered to every individual in the IDIs. The interview moderator piloted tested the key informant guide and FGD guide on 5 and 8 individuals respectively who were not part of the final data sample. Adjustments to the flow of questions and appropriateness were made before the final data collection.

### Sample size

In this qualitative study, we did not predetermine the sample size but rather based on data saturation principle which is reached when no new insights are being collected from additional of any new respiondents as informed by the theoretical framework set forth. Therefore, fourteen patients living with HIV and hypertension and eight CHWs were included in this study.

### Participant recruitment and selection

The inclusion criteria comprised of PLWHA who were ≥ 18 years old, willing to give written informed consent and had lived with HTN for more than 3 years. We excluded participants who had pregnancy-induced hypertension (women who had this confirmed by their antenatal cards) and lived beyond 30 km of the hospital catchment area. Five participants who met the inclusion criteria did not participate in the study as they had travelled out the area for the duration of the study (3) and inability to complete the study interviews (2).

### Sampling technique

CHWswith at least 3 years’ experience in delivering health education in Nakaseke, fluent in *Luganda* (language used in the health education*)* were purposively recruited into the study. The study research team approached the CHWs following obtaining this information from the public health inspector and education officer at Nakaseke hospital. Participants with HIV and HTN were coveniently sampled from the clinic register at Nakaseke hospital based on their residence, and active utilization of the clinic services at the time of the study. Upon identification in the register, the research team with the help of CHWs invited through face to face interactions and telephone calls the PLWHA/HTN to participate in the study at the hospital by providing information about the study; to provide. Separate FGDs composed of eight participants for PLWHA/Hypertension and CHWs were conducted. Variations in representation from the different regions served by the hospital catchment area were ensured throughout the FGDs. or The FGDs were not sex-specific.

### Intervention

The CHWs provided education to the patients at the hypertension clinic using the hypertension module of a validated patient booklet, *PocketDoktor*™ [19]*.* It consists of topics such as “what is hypertension?” signs and symptoms, body organs involved, treatment, management, complications and prevention and behavioral change strategies such as dietary salt intake reduction, exercise and diet modification. Different picture illustrations with complementary text written in the layperson’s terms are a major feature of the *PocketDoktor*™. The chronologically organized *PocketDoktor*™ booklet’s interactive features are also designed to stimulate patients to engage in conversations with their health educators. In Uganda, the *PocketDoktor*™ has been previously translated from English to *Luganda*, the local language as part of a previous study [19]. CHWs underwent four training sessions on how to use the *PocketDoktor*™, ethics of communicating with participants, rationale of the study, hypertension diagnosis and management practices in the laypersons’ prose and language. The intervention, part of a parent study, was delivered to each individual at the end of their routine clinic visit, with the length of sessions varying from 20 to 30 min. In-depth interviews were conducted with participants on the same day to determine their acceptability of the intervention.

### Data collection procedures

A Ugandan male medical doctor, trained in qualitative research data collection, conducted the in-depth interviews after obtaining written informed consent from the study participants. On average the IDIs lasted 35 min which is within the recommended duration of similar interviews [29]. We conducted all IDIs using and Indepth interview guide (Additional file 1) and FGDs in *luganda*, the most commonly spoken local language. We started with FGDs that gave us group-based insights about the underlying issues, which we followed up in the IDIs. Individuals who provided more information in the FGDs were further interviewed in the IDIs. All the CHWs participated in both the IDIs and one FGD. A moderator using the FGD guide (Additional file 2) conducted each eight-participant focus group discussion (FGD), with a note-taker present to take field notes. We conducted the FGDs until reaching saturation. All IDIs and FGDs were audio-recorded and conducted at the clinic. No transcripts were returned to the study participants after data collection and transcription. The interviews were conducted only in the presence of the participants and researchers.

### Qualitative data analysis

A third-party professional linguist transcribed and translated the IDIs/FGDs from Luganda into English. Back translation was done to ensure that the quality and accuracy of the translated text. All data in the transcripts were coded using de-identified respondent identification numbers. The interviewer cross-checked interview transcripts. Data analysis was a continuous and iterative process guided by thematic analysis [13]. An open coding procedure was used to assign meaning to a portion of the phrases from the transcripts. Themes and subthemes from the related codes were developed following an inductive approach. The entire interview was the unit of analysis. Interviews were reread a couple of times to get familiar with their content. Words, sentences or paragraphs that relayed a similar message were grouped as meaning units, which were then condensed and labelled with a code. I aggregated similar codes to form categories. Categories were made to be mutually exclusive, whenever that was possible and to include all the information related to the content area being discussed. Categories were further analysed to form sub themes and themes from their latent meanings [30, 31]. No apriori themes were used in the analysis. Themes were then categorized under domains of Sekhon’s Theoretical Framework of Acceptability (TFA) [28] with further classifications as either barriers of facilitators for the implementation of the intervention. The findings of the FGDs were corroborated and triangulated with the themes obtained from the in-depth interviews. We used Nvivo 11.0.0 (QRS International, Cambridge, MA) to organize the analysis process. The findings of this study are reported according to the consolidated criteria for reporting qualitative studies (COREQ) as shown in Additional file 3 [32].

### Ethical considerations

We obtained ethical approval from the Makerere University School of Biomedical Sciences IRB (SBS 610, dated 9th October, 2018) and the Uganda National Council of Science and Technology (SS # 4899). Administrative approval was sought from the Nakaseke Health Centre IV management team before commencement of study activities. All participants gave written informed consent before conduct of the in-depth interviews or FGDs. The study participants were given 20,000 Ugandan Shillings (approximately 7 USD) as a transport refund and or as a compensation for their time in the study.

## Results

We conducted 22 in-depth interviews (IDIs); 14 with PLWHA and HTN and 8 with CHWs. We also held 3 FGDs, 2 with PLWHA and HTN and one with CHWs. In this qualitative study, the average age of the PLWHA that participated in the in-depth interviewees was 55.9 years. The average duration of living with HIV and hypertension was 4.6 years. The majority (72.7%) of the PLWHA had primary level of education (Table 1).

**Table 1:** Summary of the social demographics of PLWHA and hypertension involved in the acceptability of patient centered education

Characteristics	Number (%) or average
**Sex**	
Female	27 (81.8)
Male	6 (18.2)
**Level of education**	
No formal education	3 (9.1)
University level	2 (6.1)
Primary level	24 (72.7)
Secondary level	4 (12.1)
**Occupation**	
Housewife	6 (18.2)
Peasant farmer	17 (51.5)
None	1 (3.0)
Self-employed	8 (24.2)
Student	1 (3.0)
**Marital status**	
Cohabiting	2 (6.1)
Married	6 (18.2)
Widowed	6 (18.2)
Divorced	3 (9.1)
Single	16 (48.5)
Age in years [mean (sd)]	55.9 (9.4)
Median monthly income in Uganda shillings	115,000

*sd* standard deviation

The community health workers’ age ranged between 25 and 59 years. Four were females and four were males, all had a minimum of primary level education and minimum of 3 years’ experience as CHWs in Nakaseke area. The primary level corresponds to 1–7 years of education, the secondary level to 8–13 years of education and the tertiary level or university level to more than 13 years of education.

### Acceptability of patient centered education delivered by CHWs

The section below presents the facilitators and barriers to the acceptability of CHWs in delivery of patient centered education on hypertension, organized under Sekhon’s framework (Table 2). It presents the emerging themes that were common between the FGDs and IDIs of CHWs and patients with their relevant quotations. The themes were interrelated and often overlapped. Therefore, they are not presented separately.

**Table 2:** Acceptance of patient-centered education delivered by CHW. A summary of the themes and Sekhon’s TFA construct

TFA construct	Subthemes	Facilitator or barrier status
**Affective attitude**	Rapport already exists	Facilitator
	CHW uses a non-technical language	Facilitator
**Self-efficacy and burden**^**a**^	Highly motivated	Facilitator
	Inaccessibility of the health facility	Barrier
	Financial stress	Barrier
**Perceived effectiveness and intervention coherence** ^**a**^	Potential health outcomes	Facilitator
	Understandable material	Facilitator
	Difficulty in keeping up with the technical details in *PocketDoktor™*	Barrier
**Opportunity costs and Ethicality**^**a**^	Health is a priority	Facilitator
	Availability of time	Facilitator

^a^quotes and sub-themes in these constructs overlapped and thus presented jointly

### Affective attitude

Affective attitude denotes how an individual feels (either negatively or positively) about the intervention. Four themes were identified as facilitators of affective attitudes towards the health education delivered by CHWs. These included: 1) easy-to-understand, non-technical terminology, 2) rapport already exists 3) health as a priority, 4) Availability of time.

#### “Easy to understand, non-technical terminology”

A main facilitator, highlighted by PLHWA/HTN in the IDIs and FGDs, was the CHWs’ use of colloquial terminology, translating the often-cryptic medical jargon into more laymen’s terms. Their language was expressed as easy to understand and allowed for the asking questions, which facilitated understanding. One participant stated:

*“...the health education by community health workers is easy to join and understand. I have also read the entire sensitization book they gave us…” (Interviewee 3, PLWHA/HTN)*

In addition, and likely due to this cordial, non-technical exchange, participants found the CHWs to be more approachable than with the medical doctors, where a hierarchy can sometimes be felt. Participants described a sort of affinity to the CHWs. Sharing similar lifestyles appeared to aid in their mutual understanding of one another and daily constraints in terms of living productive lives.

*“The community health worker can be able to meet a patient at all times and he can have the time unlike the doctor. …because the community health workers are in the community, they are easily reached, they are known and the patients know which time to reach the health workers” (Interviewee 21, PLWHA/HTN)*

#### Rapport already exists

PLWHA/HTN described having pre-existing interactions with CHWs who provided the health education such as having met them in previous community health promotions like immunization, education about malaria prevention, and distribution of insecticide treated nets implemented in the area. This would give them an opportunity to learn of existing health interventions. Some participants felt that they had a strong link to the health system and this facilitated their learning. When they were not at the health facility, the CHW would alert them to join for their benefit.

*“Sometimes they (CHWs) call us on phone, for example I came when I did not know that there was a teaching about pressure going on. If they are not in place, some of us stay in the villages and we do not get information.” (FGD -1, PLWHA/HTN)*

### Self-efficacy and burden

The next two themes within the Sekhon et al. model were self-efficacy and burden. Self-efficacy is an individual’s confidence that they can perform the procedures of the intervention while burden is how much effort an individual thinks is needed for the successful outcomes of the intervention if they participate. For this analysis, the sub-themes for self-efficacy and burden constructs overlapped and are reported jointly. CHWs reported high motivation to carry out the intervention. Similar findings were noted in the PLWHA and hypertension. However, challenges like financial stress, inaccessibility of the health facility, patient questions beyond skill level were barriers to their participation.

#### Highly motivated

CHWs were determined to deliver the health education to the PLWHA and hypertension. The motivators for their dedication to their work included flexibility of their roles and passion to serve their communities. They viewed this as an opportunity to extend their contacts with the community. They found value in their work and wanted to do more work for the good of the community despite being volunteers.

*“the time I spend teaching patients does not bother me because I am doing something I like and anything concerning health concerns me too because I also have my people so when I see a person who has that disease, I just think that even one of my people might be having the same. Their fore it does not bother me at all because I do the work with determination and love” - (FGD 3, CHWs)*

*For the* PLWHA/HTN, they were willing to undertake the recommendations in the intervention and felt invigorated to implement the learned knowledge.

“*We have spent around 30 minutes or more learning about hypertension. It does not burden me because the moment you come for training or drugs to stay healthy then you have to commit yourself to do that so that you stay healthy. When the health worker taught me, I got energy and courage to know that if I swallow those drugs, I get better and even the blood pressure reduces” (interviewee −7, PLWHA/HTN)*

#### Financial stress

As with voluntary work, some CHWs were stressed by the ability to meet both the financial obligations elsewhere and work at the health facility. Consequently, the CHWs were doing many other unrelated jobs, which affected the ability to perform their duties. In some cases, they would reduce how many patients they saw due to burn out or felt inconvenienced by the lengthy patient interactions. As one told us:

*“you know we have to develop ourselves; we have families and people relying on us. I am a farmer and this time I am usually in the garden...sometimes you can first do your work then you go and meet your clients. You plan accordingly, because some of us are farmers if it is time to work you decide that instead of seeing 6 clients, let me see 1 or 2, give her 2-3 hours when you are together then leave her and go to do your personal work.” (Interviewee 14, CHW).*

Other CHWs stated that for the intervention to work best, monetary facilitation to cover expenses incurred in delivering the intervention, should be considered. Another CHW stated:

*“The community worker’s demands should be put in to consideration by the doctors because our people stay in different places and they use different names. At the hospital, she uses a different name and, in the community, she also uses a different name. In order to find her, you have to make phone calls, board and go to the community and meet the leaders to investigate until you discover that she is the one. So, you have to give us facilitation in form of transport and lunch. If you do that, we as community health workers we shall do the work properly. Then at the end I also sign for an allowance that you decide to give me”. (Interviewee 19, CHW*)

#### Inaccessibility of the health facility

The participants with hypertension and HIV strived hard to attend the training at the health facility. They explained that it was challenging for them and colleagues to attend and benefit. There were challenges in finding transportation to get to the health facility for some individuals.

*“I found it hard to move the long distances to the hospital today. However, if people (we) are mobilized in their villages and sensitized (given patient education on hypertension) there, this sensitization can be made better”. (Interviewee 21, PLWHA/HTN)*

Among the CHWs, provision of the health education at the hospital led to new paradigms in their work. They felt that the hospital teaching provided less opportunity for follow up and preferred delivery of the intervention in the villages close to where they live.

*“The challenge I see is with how we can reach the hospital and the communities where these patients are because you find that they do not stay in the area where you stay meaning that you need to go to the places where they stay” – (interviewee 12, CHW)*

One of the CHWs described that the health facility setting led to reduced disclosure of concerns from patients during education. They preferred the community household setting where they would be able to interact freely with participants and answer all their questions.

*“It is better to teach patients from home than to teach them from the hospital because at home a person is so free, has a lot of time and opens up to you because she knows you”. (Interviewee 14, CHW)*

#### Patient questions beyond CHWs skill level

Sometimes CHWs were mistaken as medical doctors. As the participants gained confidence in them, they inquired into what was perceived as more detailed or complicated questions related to hypertension, which often were beyond the scope of the *PocketDoktor*™. Thus, the CHWs felt uncomfortable and unable to help them.

*“During the education session sometimes, the patient reaches a point and challenges me. They don’t know that I am just a community health worker but they refer to me as a medical doctor. Therefore, I find ways of responding so that I am not embarrassed and send them to the doctor. …. because (some aspects of) the book (are) not comprehensive (for a CHW)”. (Interviewee 13, CHW)*

### Perceived effectiveness and intervention coherence

Sekhon et al. define perceived effectiveness construct as the extent to which an intervention is expected to achieve its purpose [28]. On the other hand, intervention coherence is the extent to which participants understand the intervention and how it works. The quotes and subthemes under perceived effectiveness and intervention coherence overlapped and are thus presented jointly. Overall CHWs and PLWHA/HTN regarded health education as important for their community and lives. Despite the PLWHA/HTN understanding majority of the content of the health education, the CHWs reported difficulties staying abreast with all the technical details in the *PocketDoktor™*.

#### Potential health outcomes

PLWHA and hypertension considered the health education as beneficial to their wellbeing. They shared the view that the knowledge acquired in the session, if put into practice, would bring their blood pressure to towards normal and improve their drug adherence and psychological wellness as they live with chronic hypertension.

*“I think it might bring about a change in my blood pressure. I was diagnosed last year but my blood pressure has been persistently high. However, now that I have started getting these sensitizations, I hope to see the difference. I have understood how I should live with this illness”. (FGD 2, PLWHA/HTN)*

Based on their knowledge of the previous successful interventions, CHWs echoed the importance and future impact of the health education of the patients with hypertension. The health education would improve the tool kit for patients to live healthy lives.

*“It will improve the patients’ health. They will be able to get guidelines on what to do, reduce or stop the intake of dangerous substances and know when to go to hospital for their blood pressure symptoms”. (Interviewee 14, CHW).*

#### Understandable materials: the PocketDoktor™ book is simple to use and read

PLHWA expressed that the *PocketDoktor*™ facilitated learning and being that it was written in the local language, Luganda, it was comprehensible. The picture illustrations in the *PocketDoktor*™ provided appropriate visualizations of the causes, disease process, complications, and possible action points. It also gave them opportunities to review what was taught by the CHWs. One participant below illustrates this:

*It is good they gave us this book (PocketDoktor™ ). The pictures show you what to do. Because here the picture shows a blocked vein, so if the vein blocks that is how you start getting difficulty in breathing. When you get to that level, you are almost getting paralyzed. ..previously the doctor has been telling me some things and I reach home when I have forgotten some. I am going to start checking in the book. The good thing is that it was written in Luganda (local language), and even a primary two dropout can read Luganda and know what to do. (Interviewee 4, PLWHA/HTN)*

Even among those with poor vision, they were willing to ask their children or someone else at home to read to them the contents of the *PocketDoktor*™

*“...my eyes have poor vision; I can see some words. I can read the big letters but I cannot read the small ones. I have a child at home who can read for me.” (Interviewee 11, PLWHA/HTN).*

Six of the eight CHWs experienced health education of patients with the pocket doctor as easy to do and convenient compared to teaching without job aids. It did not require a lot of extra search for information on hypertension and too much rehearsal. It was also simple to explain to prospective clients using the booklets. The notion was expressed in both in depth interviews and the FGDs. An excerpt from the FGDs:

*“I see this book (PocketDoktor*™ *) in this way. When you open this book, the way it was written is simple in that even someone who does not know how to read can be able to understand most especially if I first read for them and explain the content. I feel confident and I do not get any fear because I see that what I am teaching even if I just explain, it contains pictures that can inform a person in a simple way”- (FGD-3, CHWs).*

#### Difficulty in keeping up with technical details

In some cases, the CHWs involved in the implementation of *intervention felt aspects of the PocketDoktor™ was too technical* and not detailed enough for their understanding. They mentioned pictures showing the blood flow and the relationship to how hypertension arose as some concepts, which made their explanations to patients quite difficult. One CHW captures this theme in the following:

*The book is not so difficult but they should do some improvement most especially where they did not elaborate well. Remember I am not a medical doctor so when I am explaining to the patient, there are some questions she might ask yet I am not well informed. (Interviewee 19, CHW)*

Furthermore, some patients reflected that the CHWs had gaps in knowledge of all the management and symptoms of hypertension. In some instances, when they asked questions related to what they found in the book and they did not receive the appropriate answers. Others felt that the health education given by CHW alone was insufficient; they needed a more trained medical doctor available to join them because of the complexity of the disease.

*“I asked CHW and told her, they said I have excess fats and they have blocked my veins, what am I supposed to do? she said that she did not know what I can do”. (Interviewee* 10, PLWHA/HTN)

### Opportunity cost and ethicality

Opportunity cost is defined as the potential loss or gain from other alternatives when a choice is made while ethicality is the extent to which the intervention is considered as a good fit with their values [28]. For these two constructs, the sub-themes overlapped and are presented together. Both CHWs and PLWHA/HTN were willing to forego some activities in order to provide or receive health education regarding hypertension. They viewed health as a priority. For the CHWs, delivering the health education rhymed with their values and had more time to teach the patients.

#### “Health is a priority”

All the people with PLWHA and hypertension viewed health education as important and felt empowered to get information about hypertension and their health. They hoped that by acquiring more information, it would allow them to control their disease process and know what actions to take for their illness.

*“I have to study and know the status of my health. Because whenever you understand your situation, it is easier, that is why they say that “fore told is fore warned”. If you remain in darkness, that situation does not go away”. (Interviewee 1, PLWHA/HTN)*

To achieve the desired changes from the health education, the study participants were willing to undertake the recommendations learnt. Some reported that they would start implementing new behavioral changes while few of them were to strengthen existing protective lifestyles.

*“I can change all the behaviors like smoking, drinking alcohol that the health worker talked about. Because previously, I have been doing them not knowing that they are dangerous”. (Interviewee 20, PLWHA/HTN)*

#### Availability of time: “the CHW has more time to explain illness”

Participants reported that education provided by the CHWs was at an appropriate pace for their age and health needs. They were given enough time to comprehend the content and ask questions to the CHWs unlike the sessions where the medical doctor was either in a hurry, too busy and had no time to teach them about their disease. As one participant noted:

*“we conversed for a long time (with the CHW) and I understood each and everything he taught me. Because the other medical doctor is always engaged with a lot of duties, he teaches you a little.” (Interviewee 1, PLWHA/HTN).*

Receiving health education about hypertension for the first time was common among the participants (18 out of the 22 who had IDIs). They would attend the clinic, queue, pick medicines, and return to their homes. The few participants, who had been given prior health education, had only been taught about adherence to the anti-hypertensive medications. Little or no information had been given about the lifestyle modifications needed when one has hypertension.

*“...ever since they told me that I have high blood pressure, today has been my first day to study (be sensitized) and I have enjoyed. It has been so good. I usually come, queue, see the clinician, pick my medicines and go home”. (Interviewee 3, PLWHA/HTN)*

Amidst the high clinic attendance numbers, the community health workers viewed themselves as complementary to the medical doctors. They viewed their health education of patients as an avenue to reduce the overall time spent in the clinic. The work of the doctors will be lighter as two CHWs told us:

*“...the doctors have various tasks to fulfill and they do not have time to teach. However, if we teach these people, the work of medical doctors becomes easy. When the patient is not given health education about hypertension and they go to ask a question, the doctor spends a lot of time on one patient. But if we teach the patient from this side, the doctor gets enough time to work on all the patients”” (Interviewee 13, CHW).*

## Discussion

This study explored the acceptability of delivering a patient-centered education for hypertension among PLWHA by CHW in Nakaseke, Uganda. The facilitators of the patient centered education were- CHWs used a non-technical language, used understandable material, had existing rapport with participants and had more time to educate the participants. Importantly, patients considered their health as a priority and expected better potential health outcomes. Difficulty in keeping up with the technical details in the *PocketDoktor*™, financial stress, patient questions beyond skill level, and accessibility of the health facility were barriers to delivery of patient centered education by CHWs.

Our study findings of facilitators of patient education by CHWs indicate that patient-centered education can be used to foster communication between health care workers and patients. CHWs were perceived as a strong link for patients to the health system. In addition, this methodology may improve medication adherence, decrease hospitalization, enhance patient involvement in medical decision-making and affect positive changes in health habits for patients with chronic diseases in low income and high-income settings [33–37]. These findings are similar to prior work in Uganda concerning implementation of patient-centered education in other hospital settings [19], which demonstrated improved patient activation, a measure of attitudes toward confidence, knowledge and ability to self-managing health. Although scale-up of HIV programs across Uganda has been largely successful, our findings emphasize the need for improved HTN education among this population where the lack of knowledge on hypertension has been documented previously [38].

Several studies note that CHWs level of knowledge is an important factor in the success of an intervention [39, 40]. Some CHWs in our study reported experiencing difficulties keeping up with the technical details in the *PocketDoktor*™ related to hypertension. Comparable challenges were noted in other studies involving CHWs delivering interventions [41, 42]. For example in Nigeria, community extension health care workers (same as CHWs) tasked with delivery of family planning education to communities had limited knowledge of the contraceptives such as emergency contraceptives, intrauterine devices (IUDs) [42]. The inadequacies in knowledge by CHWs were also reported by the PHLWHA and hypertension. A finding that may led to inaccurate passing of preventive and disease management strategies to communities. This finding has also been reported in a qualitative study evaluating community perceptions towards CHWs’ roles in HIV, Tuberculosis and hypertension in western Kenya [23]. In our study, this may be attributed to CHWs’ low level of formal education, less elaborate training material, short training durations and complex topics that may need more time for content mastery.

One of the barriers highlighted during the delivery of patient education by CHWs was the financial stress they encountered. The lack of financial support creates short falls in the coverage of childcare, food costs, lost wages, and fuel for transportation to hospital or community members’ families during health promotion activities. This may affect their work in the long term. Prior studies involving CHWs have found remuneration as a challenge [41, 43, 44]. The voluntariness of their work with incentives provided in specific tasks may further compound this problem. According to the Ugandan Ministry of Health, CHW incentives may be given during immunization campaigns, distribution of insecticide treated nets among other activities. The incentives may include monetary allowances, bags, T-shirts, certificates, high quality training and supervision [26]. This framework is based on the notion that CHWs leverage the existing health care delivery infrastructure, such as health centers and community health promoters. Conversely, a recent study in central Uganda found that provision of non-financial incentives and support supervision to CHWs improved CHW performance [45].

In our study, patients asked the community health workers questions that were perceived by the CHWs to be beyond their skill level. Given the CHWs roles as local health representatives, community members often expect assistance in other aspects of care as the result of reciprocity and trust in them [46, 47]. This form of recognition posed challenges to them as they were asked questions beyond their scope of knowledge and competence. Likewise, a similar challenge was found in a study where CHWs were involved in home based management of fever interventions in Uganda [48]. In this study, caretakers expected the CHWs to have a variety of other drugs to treat common childhood diseases in addition to malaria. Therefore, our findings, underscore the fact that community workers should be part of the multi-disciplinary team of care providers while providing the tasks they are best suited to do.

The ability of patients and health care providers to reach health facilities is integral to the provision of health care to individuals. Some PLWHA and hypertension in our study struggled to get to the health facility while a few CHWs preferred to receive education delivery at home rather than at health facility. This is at least in part due to long distances, poor road terrain and unavailability of means of transport. In a low-resource rural setting and region of high poverty levels, it raises the question of whether such interventions as ours would be better conducted in the communities at the household or village level. This is in agreement with previous studies, which have documented accessibility as a barrier to provision of healthcare services in low-income countries [49, 50].

### Limitations

Despite collecting perspectives from both CHWs and PLWHA and Hypertension who are directly impacted by the health education intervention, it is noteworthy that our study had some limitations. Our FGDs and IDIs had disproportionately higher numbers of females than males. Sex differences may affect perceptions and acceptability of interventions; this was not explored. Additionally, the fact that community health workers reside in the communities with PLWHA, some participants may have responded favorably which may bias our results. In addition, we purposively chose respondents who may not truly represent the entire views of the wider population from which they came from. There may be other opinions that were not captured but are important for the implementation of patient centered education delivered by CHWs. Finally, given that this is a single site study, the range of generalizability of our findings to similar settings in rural LMICs where CHWs participate in health programs is limited. The findings represented only the community studied.

## Conclusions

Hypertension and CVD patient-centered education delivered by CHWs using the *PocketDoktor*™ was acceptable to PLWHA and hypertension in Nakaseke area, rural, Uganda. Patients appreciated CHWs’ simple language and material, rapport, and adequate time allocated to the education sessions. However, CHWs faced financial difficulty and felt inadequate to answer a couple of technical questions. Patients also demanded for services beyond hypertension and HIV care. These findings provide preliminary evidence of the acceptability of task shifting educative tasks to CHWs in the management of hypertension but emphasize the importance of incorporating these interventions in the broader service delivery system to promote sustainability. The wider service delivery systems could provide facilitation and integrate hypertension education in the wider public health information strategy. There is need for further studies to determine the cost implications of delivering this intervention among PLWHA on a large-scale in LMICs.

## Supplementary Information

**Additional file 1.** Key informant interview guide.

**Additional file 2.** Topic guide for focus group discussions.

**Additional file 3.** COREQ: Consolidated criteria for reporting qualitative research: a 32-item checklist for interviews and focus groups.

## Data Availability

The datasets used and/or analysed during the current study are available from the corresponding author on reasonable request.

## Abbreviations

PLWHA: People Living with HIV/AIDS
CHWs: Community Health Workers
FGDs: Focused Group Discussions
CVD: Cardio-Vascular Disease
LMICs: Low- and Middle-Income Countries
IDI: In-depth Interviews
HTN: Hypertension
