# Supplementary material for: Acceptability of patient-centered hypertension education delivered by community health workers among people living with HIV/AIDS in rural Uganda
Source: BMC Public Health. 2021 Jul 7;21:1343. doi: 10.1186/s12889-021-11411-6 (PMC8264981; doi:10.1186/s12889-021-11411-6)
Supplement: Supplementary file 1 — Additional file 1. Key informant interview guide. [file 12889_2021_11411_MOESM1_ESM.docx]

# APPENDIX 1: Key informant interview guide

Good morning/afternoon

Dear sir or Madam,

You are welcome to this discussion. My name is ____________________________ and my colleague (recorder) is ________________________________

We are from Makerere University College of Health Sciences which is located in Kampala, Uganda. We are grateful to you for having accepted to be here today with us. We would like to have a chat with you about your opinions on the acceptability of patient education about Hypertension as delivered by Community Health workers among people with HIV illness. During the meeting we will solicit opinions regarding appropriateness, discontinuation, satisfaction, facilitators and barriers to implementation of the intervention. The purpose of this discussion is to get your views and make appropriate recommendations to health care providers and Ministry of Health to regarding the integration of this intervention into the routine care. I am with my colleague on the recorder for future reference during the analysis.

1. In Uganda, we have many individuals living with HIV who also get hypertension and need continuous health education. We would like to Community Health Workers (CHW) instead of hospital doctors and nurses. What do you think about this method of patient education?
2. In your opinion, what are the benefits of Hypertension related patient education delivered by CHWs in rural communities? If yes, what are they?
3. According to you, what factors do you think may facilitate the intended use of CHWs for Hypertension centred patient education among HIV patients?
4. What is your opinion about the sustainability of CHWs in providing patient education about Hypertension among HIV patients?
5. Are there any cultural or community practices are related to patient education on Hypertension that may affect use of Community Health Workers for this intervention? If yes, how do those practices affect the program of patient education on Hypertension? (Probe further about traditions and beliefs around patient education by CHWs).
6. What suggestions do you think would increase the ability of CHWs to give Hypertension related patient education among people with HIV?

**Thank you for your time.**
