# Supplementary material for: Acceptability of patient-centered hypertension education delivered by community health workers among people living with HIV/AIDS in rural Uganda
Source: BMC Public Health. 2021 Jul 7;21:1343. doi: 10.1186/s12889-021-11411-6 (PMC8264981; doi:10.1186/s12889-021-11411-6)
Supplement: Supplementary file 2 — Additional file 2. Topic guide for focus group discussions. [file 12889_2021_11411_MOESM2_ESM.docx]

# APPENDIX 2: Topic guide for focus group discussions

Date of the FGD: ___/____/________

Village _________________ Number of attendees: _____ Language used: ___________________

Moderator’s name: ________________________ Recorder: _________________________

Start time: ________ (in 24hr format) End time: _______ (in 24hr format)

Good morning/afternoon

Dear all,

You are welcome to this discussion. My name is ____________________________ and my colleague (recorder) is ________________________________

We are from Makerere University College of Health Sciences which is located in Kampala, Uganda. We are grateful to you for having accepted to be here today with us. We would like to have a chat with you about your opinions on the acceptability of patient education about Hypertension as delivered by Community Health workers among people with HIV illness. During the meeting we will solicit opinions regarding appropriateness, discontinuation, satisfaction, facilitators and barriers to implementation of the intervention. The purpose of this discussion is to get your views and make appropriate recommendations to health care providers and Ministry of Health to regarding the integration of this intervention into the routine care. Everyone has their own opinion that may differ from the rest and we shall respect that. Feel free and give us your opinions. All information in this discussion will be kept confidential. We are tape recording and taking notes to be able to keep track of all that is being discussed, is that okay with you? (**Moderator ask for verbal consent**) Thank you very much.

In order for us to discuss freely we would like to first get to know each other, one name only.

1. In Uganda, we have many individuals living with HIV who also get hypertension and need continuous health education. We would like to Community Health Workers (CHW) instead of hospital doctors. What do you think about this method of patient education?
2. In your community, are there any challenges related to use of doctors and nurses to carry out patient related health education?
3. Do you know how anything concerning the benefits of Hypertension related patient education delivered by CHWs in rural communities? If yes, what are they?
4. In this study we are employing CHWs to deliver Hypertension related patient education about hypertension, tell us about your experiences?
5. According to you, what factors may facilitate the intended use of CHWs for Hypertension centred patient education among HIV patients?
6. What is your opinion about the sustainability of CHWs in providing patient education about Hypertension among HIV patients?
7. Are there any cultural or community practices are related to patient education on Hypertension that may affect use of Community Health Workers for this intervention? If yes, how do those practices affect the program of patient education on Hypertension? (Probe further about traditions and beliefs around patient education by CHWs).
8. What suggestions do you think would increase the ability of CHWs to give Hypertension related patient education among people with HIV?

**Thank you very much for your time and great participation**
